# The Profile of Urinary Headspace Volatile Organic Compounds After 12-Week Intake of Oligofructose-Enriched Inulin by Children and Adolescents with Celiac Disease on a Gluten-Free Diet: Results of a Pilot, Randomized, Placebo-Controlled Clinical Trial

**DOI:** 10.3390/molecules24071341

**Published:** 2019-04-05

**Authors:** Natalia Drabińska, Elżbieta Jarocka-Cyrta, Norman Mark Ratcliffe, Urszula Krupa-Kozak

**Affiliations:** 1 Department of Chemistry and Biodynamics of Food, Institute of Animal Reproduction and Food Research of Polish Academy of Sciences, Tuwima 10 Str., 10-748 Olsztyn, Poland; 2 Department of Pediatrics, Gastroenterology, and Nutrition, Collegium Medicum, University of Warmia & Mazury, Oczapowskiego 2 Str., 10-719 Olsztyn, Poland; ejarocka@op.pl; 3 Institute of Biosensor Technology, the University of the West of England, University of the West of England, Coldharbour Lane, Frenchay, Bristol BS16 1QY, UK; norman.ratcliffe@uwe.ac.uk

**Keywords:** volatile organic compounds, celiac disease, gluten-free diet, gas chromatography–mass spectrometry, solid-phase microextraction, prebiotic

## Abstract

The concentration of volatile organic compounds (VOCs) can inform about the metabolic condition of the body. In the small intestine of untreated persons with celiac disease (CD), chronic inflammation can occur, leading to nutritional deficiencies, and consequently to functional impairments of the whole body. Metabolomic studies showed differences in the profile of VOCs in biological fluids of patients with CD in comparison to healthy persons; however, there is scarce quantitative and nutritional intervention information. The aim of this study was to evaluate the effect of the supplementation of a gluten-free diet (GFD) with prebiotic oligofructose-enriched inulin (Synergy 1) on the concentration of VOCs in the urine of children and adolescents with CD. Twenty-three participants were randomized to the group receiving Synergy 1 (10 g per day) or placebo for 12 weeks. Urinary VOCs were analyzed using solid-phase microextraction and gas chromatography–mass spectrometry. Sixteen compounds were identified and quantified in urine samples. The supplementation of GFD with Synergy 1 resulted in an average concentration drop (36%) of benzaldehyde in urine samples. In summary, Synergy 1, applied as a supplement of GFD for 12 weeks had a moderate impact on the VOC concentrations in the urine of children with CD.

## 1. Introduction

Volatile organic compounds (VOCs) are carbon-based molecules that are volatile at ambient temperature [1]. Hundreds of VOCs are secreted by cells of the human body, as a result of metabolic processes. The qualitative and quantitative profile of VOCs in biological fluids can vary depending on the metabolic changes; therefore, the pattern of volatile metabolites may reflect the presence of disease [2]. Several studies showed an association between the pattern of volatile biomarkers and the presence of gastrointestinal diseases [3,4,5,6,7,8,9]. Gas chromatography coupled with mass spectrometry (GC–MS), a “gold standard” in VOC analysis, was applied to distinguish patients with diarrhea-predominant irritable bowel syndrome, Crohn’s disease, ulcerative colitis, and healthy controls [8], as well as celiac disease (CD) and irritable bowel syndrome [6]. Moreover, the effect of a gluten-free diet (GFD) on the exhaled breath was evaluated [10,11]. The great success of previous studies contributed to the tremendous progress in the development of new analytical techniques for VOC detection, such as field-asymmetric ion mobility spectrometry and selected ion flow tube mass spectrometry, successfully applied in the analysis of VOCs in gastrointestinal diseases [5,12]. Recently, volatolomics was established as a new scientific domain with significant diagnostic potential [13]. The application of the VOC analysis can be an innovative and non-invasive tool for the diagnosis of diseases, as well as for the monitoring of the effectiveness of treatment [14]. 

It is believed that changes in VOCs observed in gastrointestinal diseases are the result of the impaired fermentation activity of the gut microbiota [6]. In many clinical trials, the changes in the metabolism of bacteria were suggested as more informative than the microbiota composition itself [15,16,17]. Moreover, many of the nutritional interventions had moderate or no effect on qualitative and/or quantitative changes of intestinal microbiota; however, they had a much more prominent effect on their metabolism [14,16,17]. In the intestines, the interaction between commensal bacteria, human cells, and pathogens occurs and results in the formation of hundreds of VOCs observed in feces, urine, sweat, blood, and exhaled breath [18]. The presence of intestinal VOCs in urine, sweat, blood, and breath can be related to changes in the intestinal barrier [6], which are attributed to several gastrointestinal diseases [19,20]. The analysis of VOCs in urine has several benefits over the other biological fluids. Urine collection is non-invasive and does not cause discomfort even with multiple sampling. Moreover, the concentration of VOCs in urine is higher compared to blood, as urine is pre-concentrated in the kidney, which facilitates the detection of metabolites [21]. However, on the other hand, the pre-concentration of urine can vary within and between individuals, which should be considered as a confounding factor.

CD is a life-long gluten-related enteropathy observed in genetically predisposed individuals. The prevalence of CD is estimated for approximately 1% of the global population; however, it is suggested that many patients remain undiagnosed [22]. In addition to the intestinal (abdominal pain, diarrhea) and extra-intestinal (increased bone fractures, anemia, depression) symptoms, the dysbiosis of intestinal microbiota, characterized by lower diversity and disproportion between Gram-positive and Gram-negative bacteria [23], as well as altered intestinal permeability [20], is commonly observed in CD patients. The only approved treatment of CD is a GFD. However, in many patients, even after long-term adherence to the treatment, nutritional deficiencies and a lack of intestinal recovery are observed [24,25,26]. Therefore, there is a strong need to incorporate auxiliary therapies, including GFD supplementation, into the treatment regime of the CD, followed by an evaluation of their safety and effects.

Prebiotics, defined as substrates that are selectively utilized by host microorganisms conferring a health benefit [27], were reported to increase the absorption of nutrients [28] and to improve the histomorphological parameters of intestines [29], confirmed in a clinical trial and in vivo studies. Recently, the beneficial effects of prebiotics on several aspects of health in CD patients were reported [17,30,31,32]. Briefly, prebiotics were found to stimulate the activity of the intestinal microbiota [17], modulate the amino-acid metabolism [30], improve the fat-soluble vitamin status [31], and improve bone metabolism [32]. However, the impact of GFD supplementation with prebiotics on the VOC pattern remains to be analyzed. 

In general, the number of studies evaluating VOC pattern after nutritional interventions is limited. Therefore, this exploratory, randomized, placebo-controlled study is proposed to evaluate the effect of prebiotic oligofructose-enriched inulin intake on the profile of VOCs in the urine of children and adolescents with CD following a GFD, using solid-phase microextraction with GC–MS.

*Hypothesis*: Nutritional intervention with oligofructose-enriched inulin will improve the intestinal health of children with CD, affecting the profile of urinary VOC.

## 2. Results

**Figure 1:**
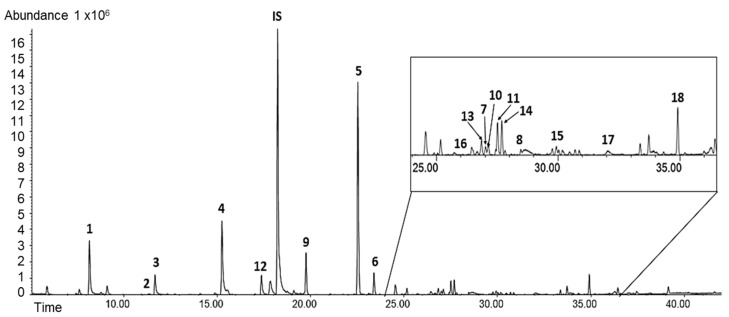
An example of a chromatogram of urinary volatile organic compounds (VOCs) obtained with gas chromatography–mass spectrometry (GC–MS): (1) acetone; (2) butane-2,3-dione; (3) butan-2-one; (4) pentan-2-one; (5) heptan-4-one; (6) heptan-2-one; (7) 6-methylhept-5-en-2-one; (8) *trans*-3-octen-2-one; (9) hexanal; (10) benzaldehyde; (11) octanal; (12) dimethyl disulfide; (13) dimethyl trisulfide; (14) d-limonene; (15) linalool; (16) 2-pentylfuran; (17) 4-methylphenol; (18) 1,3-di-*tert*-butylbenzene.

In the urine of patients with CD, a total of sixteen compounds, representing different chemical groups, were identified and quantitatively characterized (Table 1). Additionally, 4-methylphenol and 2-pentylfuran were determined in some urine samples, but values above the limit of quantification were detected only in a few samples. An example of the chromatogram is presented in Figure 1.

At baseline, the concentrations of VOCs in urine were similar in both experimental groups (Table 1). The median concentration of *trans*-3-octen-2-one was similar in both experimental groups at baseline; however, this ketone was not detected in three urine samples of patients from the Synergy 1 group.

The supplementation of GFD with Synergy 1 did not impact on the profile or the concentration of the majority of VOCs in the urine of CD patients. The only significant (*p* < 0.05) change was observed for benzaldehyde, where the concentration decreased by 36% after the intervention (Table 1). Furthermore, *trans*-3-octen-2-one was not detected in some urine samples (two from Synergy 1 group and one from the placebo group); however, it had no effect on differences between experimental groups. The decrease in the concentrations of 1,3-di-*tert*-butylbenzene was observed in the placebo group after the twelve-week intervention. 

**Figure 2:**
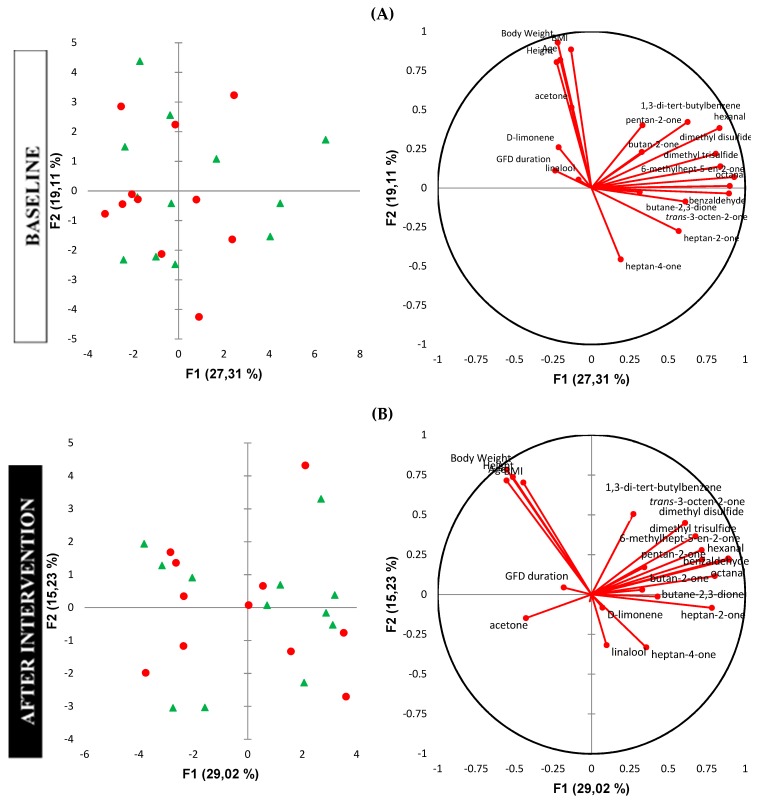
Results of principal component analysis (PCA) of urinary VOCs at baseline (**A**), and after the intervention (**B**). Red circles—Synergy 1 group; green triangles—placebo group. Left graphs—score plot; right graphs—correlation circle presenting correlations between individual VOCs and anthropometric indices.

Multivariate analysis showed a high inter-individual variation of the data (Figure 2). Principal component analysis (PCA) plots explained 46.42% and 44.25% of variations at baseline and after the intervention, respectively. No separation was observed either before or after the intervention. At baseline, anthropometric indices (age, height, body weight) had an influence on d-limonene and acetone concentrations. The level of linalool was associated with the time of GFD adherence. Similar associations were not observed after the intervention (Figure 2).

## 3. Discussion

Our study, for the first time, reports the profile and concentrations of VOCs in the headspace above the urine of children and adolescents with CD after a 12-week nutritional intervention with prebiotics applied as a supplement of GFD.

Sixteen compounds quantified in the present study were selected based on the previous studies reporting differences in urinary VOCs between healthy children and children with CD [4,33]. We hypothesized that, after the nutritional intervention with prebiotics, the urinary profile of VOC in children with CD would be altered, as a consequence of the changes in the gut caused by prebiotics. The present study indicated, however, that applied nutritional intervention did not have a strong effect on the profile of VOCs in urine. The only difference observed after the Synergy 1 intake was a significant reduction in benzaldehyde concentration. The explanation for the benzaldehyde drop in concentration can be related to the microbiota activity. Benzaldehyde can be formed as a result of the conversion of phenylalanine by aminotransferase produced by *Lactobacillus* bacteria [34]. In our study, the amount of the precursor phenylalanine was similar in both groups before and after the supplementation [30]. However, the *Lactobacillus* count was significantly lower in the Synergy 1 group as compared to placebo [17]. This might result in a reduction in phenylalanine conversion and, consequently, in decreased benzaldehyde concentration in urine.

In the placebo group, the decrease in the concentration of 1,3-di-*tert*-butylbenzene was observed. This is particularly interesting because this compound was suggested as a marker of CD, observed only in the urine of children with CD, while it existed in none of the samples from healthy children [33]. However, the origin of 1,3-di-*tert*-butylbenzene in the human body is not clear and requires further studies. It was reported that 1,3-di-*tert*-butylbenzene is a product of radiolysis of the antioxidant Irgafos used in food packaging [35], and this is a possible explanation of its origin in urine. 

The high inter-individual variation in VOC profiles makes it difficult to demonstrate significant differences after the applied prebiotic intervention. On the other hand, it may result from the recovery of the intestinal mucosa and the reduction in intestine permeability. The recent research suggests that the perturbation in the urinary VOC profile observed in some gastrointestinal diseases may result from changes in the gut barrier [6]. Children and adolescents participating in the present study were treated with a GFD for at least six months (average: 2.9 ± 1.9 and 2.3 ± 1.2 years in Synergy 1 and placebo group, respectively), which is considered as sufficient time to restore the proper functioning of the intestinal barrier [36]. In literature, the results of the prebiotic supplementation aimed to improve intestinal permeability are inconsistent. Animal studies with non-digestible fructans confirmed beneficial histomorphological changes in the gut and intestinal barrier functioning [29]. Similarly, a randomized, double-blind crossover nutritional intervention study with inulin-enriched pasta showed modulation of circulating levels of zonulin and glucagon-like peptide 2 in healthy young volunteers, suggesting that prebiotics could be used in the prevention of gastrointestinal diseases [37]. On the other hand, many clinical trials found no effect of prebiotics on intestinal barrier functions [38,39,40]. Therefore, the unambiguous impact of prebiotics on intestinal barrier functioning require further in-depth investigation.

To our knowledge, there is only one study analyzing the VOC profiles after nutritional intervention [14], making the discussion of the present results in response to other studies a challenge. The study by Rossi and co-authors [14] referred to irritable bowel syndrome and analyzed VOCs in feces. Although impossible to compare, the results presented in this interesting paper reported that the analysis of VOCs in feces can predict responses to the nutritional intervention. As in the present study, we did not observe profound differences in urinary VOCs after the applied nutritional intervention. In future studies, it would be worthwhile to analyze the VOC profile in feces of children with CD, especially as, in our previous research, we observed significant changes in the concentration of short-chain fatty acids in the feces of children with CD after the intervention with prebiotics [17].

Despite the novel nature of this study, some limitations should be mentioned. Firstly, there was no calculation of the sample size. However, this limitation is related to the pilot type of study. Therefore, the present study should be considered as an exploratory study, providing the data for calculation of the sample size for future validation studies. A second limitation was the small number of participants, causing problems in statistical evaluation based on the high inter-individual variability. This limitation is also strongly associated with the preliminary nature of the study. Thirdly, in this study, the control of the diet was not presented; however, the control of the diet was performed using validated food frequency questionnaires [41], even though details were not presented in this manuscript.

Finally, the study presented here is focused on the targeted analysis of selected compounds, limiting the number of possible responses of a non-analyzed and unknown compound. However, the authors wanted to focus on quantitative analysis, which is missing in the literature; therefore, to calculate accurately, a limited number of compounds had to be selected. However, comparing whole metabolic profiles in the urine of children and adolescents with CD after the nutritional intervention would also be scientifically interesting; therefore, it is suggested as a future study.

## 4. Materials and Methods 

### 4.1. Chemicals and Materials

Chemical standards of acetone, butane-2,3-dione, butan-2-one, thiophene, dimethyl disulfide, hexanal, heptan-4-one, heptan-2-one, 2-pentylfuran, dimethyl trisulfide, 6-methylhept-5-en-2-one, benzaldehyde, octanal, d-limonene, *trans*-3-octen-2-one, linalool, 4-methylphenol, 1,3-di-*tert*-butylbenzene, internal standard (4-methylpentan-2-ol), and sodium chloride (NaCl, ≥99.5%) were supplied by Sigma-Aldrich (Saint Louis, MO, USA). MilliQ water (Millipore, Bedford, MO, USA) was used for the preparation of standards. Hydrochloric acid (HCl, 37%) was purchased from Chempur (Piekary Śląskie, Poland). The 75-μm carboxen/polydimethylsiloxane (CAR/PDMS) (stable flex) solid-phase microextraction (SPME) fibers were purchased from Supelco (Bellefonte, PA, USA).

### 4.2. Study Protocol

A randomized, placebo-controlled, single-center clinical trial with nutritional intervention was performed. The full details of the study protocol, inclusion/exclusion criteria, and a CONSORT chart are available elsewhere [42]. The present study is part of a larger study which was registered in the US National Library of Medicine (identifier: NCT03064997; http://www.clinicaltrials.gov). The study was performed in the Gastrointestinal Clinic of the Children’s Hospital in Olsztyn from January to June 2016. A brief description of original study is as follows: 34 children diagnosed with CD and following a GFD for at least six months were randomly assigned to a group receiving 10 g per day of oligofructose-enriched inulin (Synergy 1; Orafti^®^, Beneo, Belgium) or a group receiving placebo (maltodextrin) for a period of 12 weeks. The placebo and prebiotic supplements were identical in appearance and taste. Participants and their caregivers, clinicians, and most of the investigators (except one person providing supplements) were blinded. During the intervention, participants were asked to note any side effects and daily supplement intake. Children were under the medical supervision of a gastroenterologist, and blood morphology data can be found elsewhere [43]. 

The study protocol was approved by the Bioethics Committee of the Faculty of Medicine of the University of Warmia and Mazury in Olsztyn, Poland (decision No. 23/2015). All procedures involving human participants were performed with the ethical principles of the 1964 Declaration of Helsinki and its later amendments. Parents or caregivers of participants were fully informed about the study and signed the written informed consent on the first check-up visit. 

In the present study, urine samples collected from 23 children were analyzed: 11 children from the Synergy 1 group and 12 children from the placebo group. Patients’ anthropometric characteristics are presented in Table 2. A smaller number of samples used in this study compared to the original study were related to antibiotic intake during the intervention (two persons), inappropriate compliance (less than 80% of time) to a nutritional intervention assessed based on the intervention diary (two persons), and insufficient amount of urine provided for VOC analysis (seven persons). Fresh morning urine samples were collected from each participant at baseline and after the intervention. Samples were immediately centrifuged at 3500 rpm for 10 min, and aliquots of 4 mL were stored at −80 °C until further analysis. 

### 4.3. VOC Analysis

Analysis of VOCs in urine was performed according to the previously published protocol [33]. Briefly, 4 mL of urine was placed in 20-mL headspace vials with 2.98 g of sodium chloride and 21 µL of 6 M hydrochloric acid. Then, 4-methylpentan-2-ol was added to each sample as an internal standard with a concentration of 196.24 nmol/L. Samples were incubated for 20 min at 30 °C with a shaking speed of 500 rpm using a MultiTherm shaker (Benchmark Scientific, Edison, NJ, USA), resulting in the release of VOCs from urine and their accumulation at the headspace. Next, the previously conditioned CAR/PDMS fiber was manually inserted into the headspace, and extraction was carried out for 15 min at 30 °C. After extraction, the fiber was introduced into the gas chromatography injector port with a 0.75-mm inner diameter (ID) splitless glass liner (Supelco, Bellefonte, PA, USA), set to a splitless mode, with an inlet temperature of 240 °C. Thermal desorption was carried out for 10 min to avoid carryover. 

Analysis of VOCs was performed using an HP 5890 gas chromatograph coupled with an HP 5972 mass selective detector (Agilent Technologies, Santa Clara, CA, USA) [33]. The compounds were separated using a Zebron ZB-624 capillary column, 60 m × 0.25 mm × 1.40 μm (Phenomenex, Torrance, CA, USA). The carrier gas was helium at a constant flow rate of 1 mL·min^−1^. The oven temperature program was set as follows: 40 °C for 2 min, an increase to 220 °C at a rate of 5 °C∙min^−1^, and maintained at the final temperature for 5 min. Total run time was 42 min. Mass spectra were obtained by electron ionization (EI) in the range of 40–550 *m*/*z*, and a solvent delay was set for 5 min. Ion source temperature was 230 °C and electronic impact energy was 70 eV. Total ion chromatograms were analyzed with the MSD ChemStation E.02.02.1431 software (Agilent Technologies, Santa Clara, CA, USA). Identification of compounds was performed by comparison of the retention times and mass spectra to commercial standards. Quantification of compounds was done by external standard calibration, and the results were normalized relative to the peak area of the internal standard. The previously described method was extended for analysis of acetone, pentan-2-one, d-limonene, and linalool. The content of pentan-2-one was calculated, using heptan-2-one as the external standard, by applying the arbitrary response factor of 1.00. For all compounds, the calibration curves were prepared in the same way as described previously [33].

### 4.4. Statistical Analysis

All analyses were performed in duplicate. The normality of the quantitative variables was evaluated using the Shapiro–Wilk *W* test. The comparison of anthropometric indices at baseline between Synergy 1 and the placebo group was performed using a parametric Student’s *t*-test. As the VOC data showed non-normal distribution, quantitative variables were expressed as median values (P25–P75). Differences in the concentration of individual VOCs between Synergy 1 and the placebo group were tested with the non-parametric Mann–Whitney *U* test. VOC concentrations within the group, before and after the intervention, were compared using the Wilcoxon signed-rank test. Results were considered statistically significant at the 5% critical level (*p* < 0.05). Exploratory data analysis using PCA was carried out to interpret the complex data and to determine if the differences between experimental groups could be seen. Both univariate and multivariate analyses were performed using XLSTAT for Excel software.

## 5. Conclusions

In summary, this pilot study indicated that oligofructose-enriched inulin, applied as a supplement of GFD for 12 weeks, had a moderate impact on the concentrations of VOCs in the headspace above the urine of children and adolescents with CD. It is possible that the prolongation of the study may result in a more dominant effect. Further studies are needed to confirm the effect of prebiotics on gut integrity and, consequently, on the profile of VOCs in different biological fluids. Moreover, the origin of the VOCs in the human body requires further examination.

## Figures and Tables

**Table 1:** Volatile organic compounds (VOCs) (nmol/L) detected and quantified in the urine of children from Synergy 1 and placebo group, before (T0) and after (T1) the intervention, expressed as median (P25–P75).

	T0		T1	
	Placebo	Synergy 1	Placebo	Synergy 1
Ketones				
acetone	12023 (9066–17649)	12184 (10229–17740)	12816 (10549–14704)	12564 (9969–19006)
butane-2,3-dione	66.80 (50.50–88.74)	63.22 (53.03–104.73)	59.10 (28.12–68.74)	53.63 (44.46–65.58)
butan-2-one	167.59 (73.60–229.55)	180.77 (95.76–271.57)	168.38 (116.26–313.56)	229.20 (126.17–294.85)
pentan-2-one	21.76 (9.42–57.88)	31.80 (20.55–54.27)	41.57 (18.19–60.49)	40.04 (32.83–64.30)
heptan-4-one	41.91 (22.65–125.91)	53.32 (28.13–87.32)	86.19 (32.29–103.91)	84.02 (51.86–130.34)
heptan-2-one	6.94 (3.42–14.82)	6.11 (2.59–17.55)	10.31 (3.39–12.98)	8.04 (5.38–11.73)
6-methylhept-5-en-2-one	1.31 (0.57–4.08)	1.33 (0.50–2.30)	1.87 (0.38–2.68)	1.33 (0.52–1.83)
*trans*-3-octen-2-one	0.59 (0.39–4.49)	0.56 (0.41–0.92)	1.08 (0.46–1.90)	0.71 (0.39–1.02)
Aldehydes				
hexanal	37.38 (24.60–59.95)	23.79 (17.99–36.84)	36.59 (24.22–45.63)	28.27 (18.84–38.78)
benzaldehyde	7.14 (3.14–22.86)	7.16 (3.47–12.94)	7.53 (2.48–10.00)	6.21 (3.52–7.14) ^a^
octanal	0.83 (0.35–4.11)	0.62 (0.15–2.58)	0.85 (0.20–2.04)	1.11 (0.39–1.34)
Sulfur compounds				
dimethyl disulfide	19.29 (11.39–23.26)	13.02 (6.86–18.44)	8.94 (6.70–15.16)	12.67 (7.02–19.29)
dimethyl trisulfide	1.22 (0.95–3.30)	1.01 (0.34–2.27)	1.28 (0.46–1.90)	1.72 (0.50–3.09)
Terpenes				
limonene	45.27 (9.29–67.86)	32.56 (4.02–42.40)	36.78 (24.22–45.63)	28.79 (5.46–62.43)
linalool	20.63 (14.68–28.12)	19.28 (15.99–29.20)	16.14 (11.86–26.20)	18.09 (11.76–26.24)
Aromatic compounds				
1,3-di-*tert*-butylbenzene	0.82 (0.42–1.25)	0.52 (0.33–0.92)	0.66 (0.34–0.87) ^a^	0.57 (0.33–0.91)

a—statistically significant differences within groups before and after the intervention.

**Table 2:** The participants’ anthropometric data. Results are presented as ranges and means ± standard deviation.

	Synergy 1 Group	Placebo Group	*p*-Value
*N*	11	12	
Gender	Girls—7; Boys—4	Girls—8; Boys—4	0.886
Age (years)	5–18; Av^1^ = 10.8 ± 4.1	4–16; Av = 10.2 ± 4.4	0.720
Body weight (kg)	15.8–67.9; Av = 38.3 ± 16.9	16.3–66.8; Av = 35.6 ± 17.0	0.703
Height (m)	112.5–170.0; Av = 145.1 ± 21.3	103.0–172.0; Av = 139.4 ± 22.6	0.540
BMI (kg/m^2^)	12.5–23.5; Av = 17.2 ± 3.7	13.7–28.4; Av = 17.3 ± 4.0	0.962

^1^ Av = average; BMI = body mass index.
